# Applicability of the real ear measurement for audiological intervention of tinnitus^☆^^☆☆^

**DOI:** 10.1016/j.bjorl.2018.07.010

**Published:** 2018-08-23

**Authors:** Andressa Vital Rocha, Maria Fernanda Capoani Garcia Mondelli

**Affiliations:** a Universidade de São Paulo (USP), Faculdade de Odontologia de Bauru (FOB), Bauru, SP, Brazil; b Universidade de São Paulo (USP), Departamento de Fonoaudiologia, Bauru, SP, Brazil

**Keywords:** Tinnitus, Hearing loss, Hearing AIDS, Counseling, Zumbido, Perda de audição, Aparelhos de amplificação sonora individual, Aconselhamento

## Abstract

**Introduction:**

Tinnitus is present in a large part of chronic health complaints, and it is considered a public health problem injurious to the individual's quality of life. Considering the increase of the world population associated with an increase of life expectancy, tinnitus remains a cause for medical concern, since during aging the occurrence of auditory impairments due to the deterioration of the peripheral auditory structures and central impairs the quality of life.

**Objective:**

The aim of the present study was to analyze the applicability of real ear measurements for audiological intervention of tinnitus through specific evaluation, selection, verification and validation of the hearing aids combined with the sound generator.

**Methods:**

Forty individuals of both genders with hearing loss and tinnitus complaints were deemed eligible to compose the sample. They were enrolled according to clinical symptoms and submitted to the following procedures: anamnesis and previous complaint history, high frequency audiometry, immittanciometry and acuphenometry with the research of psychoacoustic thresholds of pitch, loudness and minimum masking threshold, sound generator, in addition to the application of the Tinnitus Handicap Inventory and Visual Analog Scale tools. The entire sample was adapted with Siemens hearing aids and a sound generator, participated in a counseling session with support of digital material and evaluated in two situations: Initial Assessment (before the hearing aids and sound generator adaptation) and Final Assessment (6 months, after adaptation). The statistical analyzes were descriptive and inferential, adopted a significance level of 5% and the T-Paired Test and the Spearman Correlation test were performed.

**Results:**

The results showed that there was a benefit with the use of hearing aids combined with a sound generator from the statistically significant values and strong correlations between the sound generator verification data regarding acuphenometry and the nuisance/severity questionnaires. Regarding the verification of the sound generator, it is important to highlight that the entire sample selected the effective acoustic stimulation based on the comfort levels, which was proved in the present study to be a sufficient intensity for positive prognosis, whereas the users’ noises were found below the psychoacoustic thresholds of acuphenometry.

**Conclusion:**

The present study concluded that the audiological intervention with any level of sound stimulus is enough to obtain a positive prognosis in the medium term. Data that specifies that the verification of sound generator was effective at the real ear measurements are important in the evaluation and intervention of the complaint. In addition, it points out that the greater the tinnitus perception, the greater its severity, and the greater the nuisance, the higher the psychoacoustics thresholds of frequency and the minimum threshold of masking.

## Resumo

**Introdução:**

O zumbido está presente em grande parte das queixas crônicas de saúde, é considerado um problema de saúde pública, prejudicial à qualidade de vida do indivíduo. Considerando o aumento da população mundial associado ao aumento da expectativa de vida, a tendência é que o zumbido permaneça como um motivo de preocupação, uma vez que com a idade a ocorrência de prejuízos auditivos decorrentes da deterioração das estruturas auditivas periféricas e centrais ocasiona grande impacto negativo na qualidade de vida.

**Objetivo:**

Analisar a aplicabilidade das medidas da orelha real para a intervenção audiológica do zumbido através de avaliação específica, seleção, verificação e validação do aparelho de amplificação sonora individual combinada ao gerador de som.

**Método:**

Quarenta indivíduos de ambos os sexos com perda auditiva e queixa de zumbido foram considerados elegíveis para compor a amostra. Eles foram atendidos conforme a demanda clínica e submetidos aos seguintes procedimentos: anamnese e história pregressa da queixa, audiometria de alta frequência, imitanciometria e acufenometria com pesquisa dos limiares psicoacústicos de *pitch*, *loudness* e limiar mínimo de mascaramento, gerador de som, além da aplicação das ferramentas *tinnitus handicap inventory* e escala visual analógica. Toda a amostra recebeu aparelho de amplificação sonora individual e gerador de som, ambos da marca Siemens, participaram de uma sessão de aconselhamento com apoio de material digital e foram avaliados em duas situações: Avaliação inicial (antes da adaptação dos aparelhos de amplificação sonora individual e gerador de som) e Avaliação final (6 meses após a adaptação). As análises estatísticas foram descritivas e inferenciais, adotou-se um nível de significância de 5% com a realização do teste *t* pareado e o teste de correlação de Spearman.

**Resultados:**

Os resultados mostraram que houve benefício com o uso de aparelho de amplificação sonora individual combinado ao gerador de som com base nos valores estatisticamente significantes e fortes correlações entre os dados da verificação do gerador de som em relação à acufenometria e os questionários de incômodo/gravidade. Em relação à verificação do gerador de som, é importante destacar que toda a amostra selecionou a estimulação acústica efetiva baseada nos níveis de conforto e no presente estudo demonstraram ser de intensidade suficiente para o prognóstico positivo, enquanto que os ruídos dos usuários estavam abaixo dos limiares psicoacústicos da acufenometria.

**Conclusão:**

A intervenção audiológica com qualquer nível de estímulo sonoro é suficiente para obter um prognóstico positivo em médio prazo. Os dados que demonstram que a verificação do gerador de som foi efetiva nas medidas da orelha real são importantes na avaliação e intervenção da queixa. Além disso, mostram que quanto maior a percepção do zumbido, maior a sua gravidade; e quanto maior o incômodo, maiores os limiares de frequência psicoacústica e o limiar mínimo de mascaramento.

## Introduction

Tinnitus impairs the quality of life of millions of individuals around the world and is associated with most hearing loss (HL), being common in chronic health conditions.^1^ It is a highly prevalent and harrowing complaint, considered a huge burden that causes great losses in the quality of life. The literature indicates aversive psychological reactions, cognitive, emotional and attentional problems resulting from this condition.^2^, ^3^

In relation to the prevalence of tinnitus, a cross-sectional study with 1960 field interviews found that 22% of the population in the city of São Paulo (Brazil), 26% female and 17% male, experience this symptom.^4^ International studies have observed that progressivity in prevalence increases as a result of age, noting that about 14% of individuals aged 60–69 years present tinnitus.^5^

Tinnitus may occur along pathophysiological changes throughout the auditory pathway since sensory deprivation leads to important functional and structural changes of the auditory system followed by functional and structural alterations of the auditory system as a whole, a negative consequence of the decrease in the peripheral input.^6^ In order to maintain neural homeostasis, a noise is generated due to the hyperactivity of the nervous structures, which may or may not be decoded, generating the perception of tinnitus.^7^

To obtain relevant diagnostic information on the psychoacoustic measurement of tinnitus, certain parameters must be inherent to tinnitus therapy to guide and select the appropriate treatment, quantify, as well as to substantiate its effects.^8^

The restoration of auditory input through the use of hearing aids (HA) has been proposed as a potentially important means in tinnitus intervention that accompanies sensorineural HL. From this restoration, the reorganized neural network that generates tinnitus must be interrupted.^9^

Sound therapy associated with proper counseling has gained wide acceptance in the audiological management of tinnitus. This therapy incorporates a wide range of device options including: ambient sound enrichment devices, recorders with music and natural sounds, HA and Sound Generator (SG) at the auditory level which employs noise through the use of bandwidth to alleviate tinnitus through masking and promote habituation.^10^, ^11^

Although it is established that intervention is necessary to minimize the tinnitus process, therapies that make use of sound stimuli are very comprehensive and there are few guidelines for sound selection as well as the application of sound therapy. Thus, considering the subjectivity of the complaint, studies are needed to guide the proposed treatment with no dependence on the patient's response, but a measure capable of assessing the noise provided by the SG and the sound therapy being applied. The most objective measure to assist these individuals during the treatment is verified by real ear measurement of the noise provided by the SG. This data aids during the entire therapeutic process and favors the final result.

Evaluation methods are available to help professionals customize sound therapy and empower patients to be active in therapeutic decision making, favoring the prognosis.^12^ Thus, the present study proposes to characterize the verification of the SG and correlate it with the evaluation data of tinnitus.

## Objective

Analysis of the applicability of real ear measurements for audiological intervention of tinnitus through specific evaluation, selection, verification and validation of the hearing AIDS combined with the sound generator.

## Methods

The present study was developed as a non-randomized clinical trial, approved by the Research Ethics Committee at CAAE: 59797816.4.0000.5417 and patient consent for voluntary participation in the study and publication of the data, confirmed by signing of the Free and Clarified Consent.

Individuals of both sexes, over 18 years of age with a complaint of bilateral chronic tinnitus and diagnosis of symmetrical bilateral mild to moderate sensorineural HL were eligible to form the study sample. They could not have performed intervention prior to tinnitus, should make effective use of HA and present good general health, being able to participate in the research, as well as respond to the questionnaires.

The study was developed in two stages: Initial Evaluation and Final Evaluation (6 months). After otorhinolaryngological evaluation, interview and reception, conventional and high frequency tonal audiometry were performed. The extended frequencies evaluated from 8 kHz were: 9; 10; 11.2; 12.5; 14; 16 and 20 kHz. The evaluation was performed in an acoustically treated room, the AAF in an acoustic cabin with the Interacoustics AC40 audiometer and HH200 headphones, both with the appropriate calibrations.

In both evaluations (initial and final), the Tinnitus Handicap Inventory (THI) questionnaire was used, it is composed of 25 questions with three options: “yes” (4 points), “sometimes” (2 points) and “no” (0 points).^13^ The higher the score the greater the damage to the quality of life of the individual.

The Visual Analog Scale (VAS) was used, and values related to the degree of annoyance caused by tinnitus were assigned. The analysis of the answers is directly proportional: the greater the annoyance, the greater the value determined by the individual. The procedure was repeated at the end of the intervention process, ensuring the understanding of the proposal to reassess the annoyance of tinnitus at the respective treatment moments.

Tinnitus was measured by means of acuphenometry, with the search for psychoacoustic thresholds of pitch and loudness similar to sensation of the individual and Minimum Masking Level (MML). This method is subjective and encompasses a set of audiological techniques to find a psychoacoustic threshold that is as close as possible to the patient's tinnitus at the moment the symptom occurs.

The patients were submitted to an individual information counseling session, in which questions related to the physiology of hearing, tinnitus and hearing pathophysiology were addressed, using simple language and supported by digital material with videos and illustrations proposed by Siemens Audiology Solutions through counseling “Counseling Suite 3.3”.

The selection, adaptation and verification of HA were performed by the researcher in accordance with the Auditory Health Instruction and the American Academy of Audiology. All subjects were adapted with the HA mini behind the ear with a fine tube, domes open, semi open and closed, the “Life 501” of the Siemens brand according to audiological characteristics, acoustic and physical needs of each participant. Hearing AIDS were offered free of charge by the public health system.

The HA programming was performed according to the HL without specific considerations through the presence of tinnitus at that time. Subjects were bilaterally adapted with HAs and SGs. In order to ensure the effective use of sound stimulation, only one program was activated and oriented for effective use for a minimum of 8 h per day, monitored by the data logging algorithm.

After the HA programming, the verification procedure of the adaptation was carried out by means of the measurements with a probe microphone, an objective method that encompasses the physical characteristics of the ear in the amplified response. Measurements with the probe microphone were performed in an acoustically treated room using the GN Otometrics’ Aurical Free Fit equipment. For this amplification verification, the prescriptive method developed by the National Acoustic Laboratories – NAL NL1 was selected.

The SG verification was also performed using the same equipment that provides an efficient way to verify the SG's feature for tinnitus management. With the OTOsuite software, the Aurical allows direct access to tinnitus data from an additional set of markers that overlaps the audiogram from psychoacoustic measurements. The markers are available in the Real Ear and 2cc Coupler modes, and are evaluated in the “Free Style” screen.

**Figure 1:**
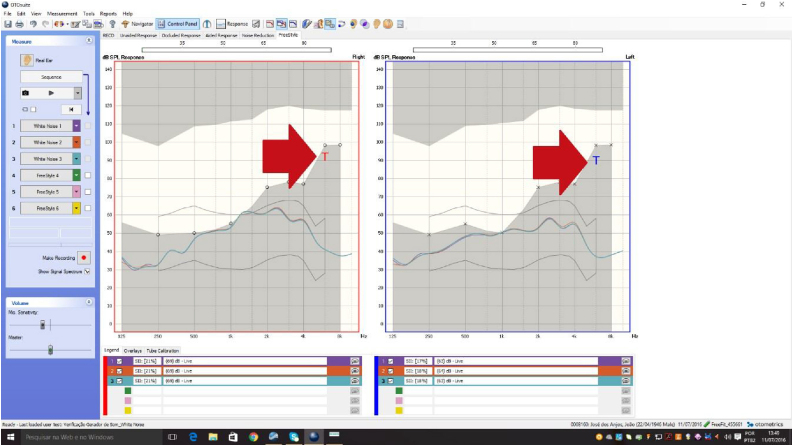
Example of the curve obtained by the real ear measurements and marking of the psychoacoustic measure (acuphenometry) as a “T” of tinnitus.

The procedure was also performed in an acoustically treated room using the equipment and standards previously mentioned. In order to verify the SG, measurements were made with a live probe microphone, an objective method that expresses the white noise curve that was provided in the external auditory canal (Fig. 1). The protocol developed by the researcher found in the software of the equipment was selected so that the curve referring to the noise was drawn.

These data assist in the orientation and in the process of adaptation and verification, resulting in a better prognosis. So far there is no standardization, characterization or any other finding that exploits these measures in literary studies, emphasizing the necessity of the development of this research.

As a result, the “noise curve” provided to the patient according to his choice of audibility and comfort point was measured, as well as being characterized and analyzed through the “SPLogram” and acuphenometry to provide intervention indicators and verify the effectiveness of the auditory stimulation proposal.

The SPLogram is a graph representing the audiometric thresholds as a function of frequency on a dB sound pressure level scale. It is different from the audiogram, which is a graph representing the audiometric thresholds as a function of frequency on a Decibel Hearing Level (dBHL).

Considering the fact that the acuphenometry was obtained in dBHL and inserted in the Otosuite software in the same way, the transcription of the thresholds for Sound Pressure Levels (SPL) through the NOAh software itself was performed. The same was possible by adding a new record for each patient and simulating an audiometry with all frequencies presenting the same thresholds and only the correct acuphenometry threshold so that a target and a SPL value for this psychoacoustic threshold were generated. Subsequently, the screen of the measurements with the probe microphone was opened and the value was measured by the program cursor positioned on the single “point” beyond the “target curve”. Both measurements were compared in dBHL and SPL, and it was observed that despite the need for transcription for data reliability, both values would lead to the same results and statistical analysis since they did not substantially modify their positions on the graph.

After 3 months of the Initial Evaluation, all patients returned to the appointments to make fine adjustments of the HA schedules according to the need of each patient. After 6 months of the Initial Evaluation, all patients returned to the visits, and the protocol pertinent to the development of the study was reapplied.

### Statistical analysis

The analysis of the results was performed based on descriptive, inductive or inferential statistics. All statistical procedures were performed in the Statistical version 10.0 software (Stat Soft Inc, Tulsa, USA), adopting a level of significance of 5%.

For descriptive analysis, the mean and standard deviation of all the numerical variables of the study were used, and the Spearman Correlation was used for inferential analysis for the correlations and the *t*-Test for the comparisons.

The diagnostic value data, which did not change during the study, were presented in a descriptive way by means of tables in the sample composition.

## Results

The sample consisted of 40 individuals of both sexes, being 24 men and 16 women with a mean age of 66.92, with a standard deviation of 8.36. Concerning the degree of HL, 17 subjects had a mild degree and 23 had a moderate degree, equivalent to 42.50% and 57.5%, respectively. The audiometric thresholds of frequencies of 0.5, 1, 2 and 4 kHz were used to classify HL according to the World Health Organization,^14^ as shown in the table below.

The predominant HL configuration was down, occurring in 77.5% (*n* = 31) of the cases and flat in 22.5% (*n* = 9). According to secondary data obtained in the medical records, the diagnostic hypothesis for all disorders was unknown etiology.

The averages of the audiometric thresholds obtained in the conventional diagnosis were 40.27 dB – with a standard deviation of 8.64 for the right ear and a mean of 40.92 dB – with a standard deviation of 8.04 for the left ear. All the audiometric thresholds obtained in the AAF (9 kHz to 20 kHz) were found below the normality standards.^15^

The results of the psychoacoustic thresholds of the acuphenometry in the evaluation stage are described in Table 1. In the Final Evaluation situation, there was no need to expose the data statistically, considering that the tinnitus complaint was no longer constant from the suppression and remission of the symptom, and the procedure was not performed in the return of all patients.

**Table 1:** Mean and SD values of the responses obtained in the acuphenometry of the initial assessment.

Acuphenometry		Mean ± SD
Frequency of tinnitus sensation (kHz)	RE	5.32 ± 2.06
	LE	5.25 ± 2.07
Sensation of tinnitus intensity (dBSL)	RE	2.12 ± 5.93
	LE	1.95 ± 6.48
Minimum masking level (dBSL)	RE	3.47 ± 5.36
	LE	3.22 ± 5.54

SD, standard deviation; RE, right ear; LE, left ear; kHz, quilohertz; dBSL, decibel sensation level.

**Figure 2:**
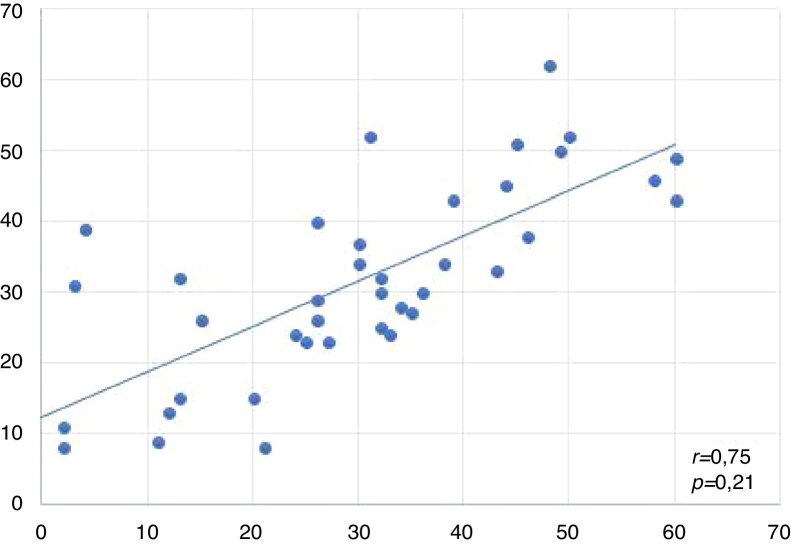
Scatter plot of the psychoacoustic thresholds of Pitch and Loudness of acuphenometry (right and left ears).

Spearman correlations between the results of the studied variables are shown in Fig. 2 as well as in Table 2, Table 3, Table 4, Table 5, Table 6. The “SG verification” or “SG curve” is said to be the difference between the threshold psychoacoustics obtained in the acuphenometry and the curve drawn by the real ear measurement at that specific frequency.

**Table 2:** Spearman correlation *r* and *p*-values between THI and acuphenometry.

Acuphenometry		THI	Total		Functional		Emotional		Catastrophic	
			*r*	*p*	*r*	*p*	*r*	*p*	*r*	*p*
Frequency of tinnitus sensation (kHz)	IA	RE	−0.16	0.321	−0.24	0.125	0.01	0.959	0.15	0.345
		LE	−0.21	0.189	−0.26	0.102	−0.03	0.843	0.01	0.945
	FA	RE	0.17	0.275	0.18	0.248	0.14	0.374	0.08	0.617
		LE	0.17	0.286	0.19	0.224	0.13	0.392	0.05	0.747
Sensation of tinnitus intensity (dBSL)	IA	RE	−0.07	0.631	−0.05	0.753	0.13	0.404	0.40^a^	0.896
		LE	0.08	0.581	0.05	0.715	0.31^a^	0.048	−0.26	0.964
	FA	RE	0.35^a^	0.235	0.39^a^	0.125	0.29	0.668	0.19	0.221
		LE	0.18	0.247	0.21	0.184	0.21	0.173	−0.02	0.884
Minimum masking level (dBSL)	IA	RE	0.02	0.855	0.09	0.565	0.03	0.843	−0.26	0.963
		LE	−0.12	0.43	−0.15	0.34	−0.3	0.557	0.24	0.131
	FA	RE	0.25	0.118	0.22	0.166	0.27	0.087	0.12	0.43
		LE	−0.18	0.252	−0.27	0.835	−0.11	0.497	−0.1	0.946

THI, tinnitus handicap inventory; RE, right ear; LE, left ear; kHz, quilohertz; dBSL, decibel sensation level; IA, initial assessment; FA, assessment final; r, correlation coefficient.

a *p* < 0.05, Spearman Correlation.

**Table 3:** Spearman correlation *r* and *p*-values between the VAS variables and acuphenometry.

VAS	Frequency of tinnitus sensation (kHz)				Sensation of tinnitus intensity (dBSL)				Minimum masking level (dBSL)			
	RE		LE		RE		LE		RE		LE	
	*r*	*p*	*r*	*p*	*r*	*p*	*r*	*p*	*r*	*p*	*r*	*p*
IA	−0.1	0.41	0.05	0.17	−0.14	0.371	−0.09	0.56	−0.1	0.53	−0.03	0.74
FA	0.07	0.61	0.08	0.64	0.44^a^	0.409	0.17	0.27	0.24	0.12	−0.18	0.26

a *p* < 0.05, Spearman Correlation. VAS, Visual Analog Scale; RE, right ear; LE, left ear; kHz, quilohertz; dBSL, decibel sensation level; IA, initial assessment; FA, assessment final; *r*, correlation coefficient.

**Table 4:** Spearman correlation *r* and *p*-values between the SG and acuphenometry variables.

	Frequency of tinnitus sensation (kHz)				Sensation of tinnitus intensity (dBSL)				Minimum masking level (dBSL)			
	RE		LE		RE		LE		RE		LE	
	*r*	*p*	*r*	*p*	*r*	*p*	*r*	*p*	*r*	*p*	*r*	*p*
SG	0.74^a^	0.265	0.63^a^	0.92	−0.04	0.766	−0.02	0.897	−0.38^a^	0.144	−0.18	0.258

a *p* < 0.05, Spearman Correlation. SG, sound generator; RE, right ear; LE, left ear; kHz, quilohertz; dBSL, decibel sensation level; IA, initial assessment; FA, assessment final; *r*, correlation coefficient.

**Table 5:** Spearman correlation *r* and *p*-values between SG and THI variables.

GS	Total				Functional				Emotional				Catastrófico			
	OD		OE		OD		OE		OD		OE		OD		OE	
	*r*	*p*	*r*	*p*	*r*	*p*	*r*	*p*	*r*	*p*	*r*	*p*	*r*	*p*	*r*	*p*
AI	−0.09	0.550	−0.17	0.280	−0.12	0.420	−0.09	0.560	0.02	0.880	−0.08	0.590	0.11	0.490	−0.08	0.610
AF	0.07	0.620	0.16	0.280	0.10	0.500	0.20	0.200	0.03	0.840	0.03	0.480	0.05	0.730	0.08	0.580

SG, sound generator; RE, right ear; LE, left ear; kHz, quilohertz; dBSL, decibel sensation level; IA, initial assessment; FA, assessment final; *r*, correlation coefficient.

**Table 6:** Spearman correlation *r* and *p* values between SG and VAS variables.

VAS	SG			
	RE		LE	
	*r*	*p*	*r*	*p*
IA	−0.11	0.46	−0.34^a^	0.28
FA	−0.09	0.54	−0.04	0.78

a *p* < 0.05, Spearman Correlation. VAS, Visual Analog Scale; SG, sound generator; RE, right ear; LE, left ear; IA, initial assessment; FA, assessment final; *r*, correlation coefficient.

### Correlation between RE/LE and acuphenometry variables

The graph below represents the correlation of the acuphenometry variable (measured only by pitch and loudness) by means of the difference between the tinnitus threshold and the peak of the curve at that same frequency in both ears.

This graph indicates that the stimulation between the ears was close, and there was a statistically significant difference between them, suggesting that the same type of sound therapy was selected on both sides.

### Correlation between THI and acuphenometry

The table below shows the correlations between THI and acuphenometry. Significant correlations were observed for all subscales in relation to the psychoacoustic measure of intensity in the initial and final evaluations.

### Correlation between VAS and acuphenometry

The table below shows the correlations between the VAS and acuphenometry variables. There was a significant correlation between the VAS and sensation of intensity in the final evaluation.

### Correlation between SG verification and acuphenometry

The table below shows the correlations between the GS and acuphenometry variables. There was a significant correlation between the GS and sensation of frequency as well as in the MML.

### Correlation between the verification of SG and the THI

The table below shows the correlations between the GS and THI variables. There was no significant correlation between them.

### Correlation between the verification of SG and VAS

The table below shows the correlations between the GS and EVA variables. There was a significant correlation between them in the initial evaluation.

## Discussion

The sample consisted predominantly of males: 24 males and 16 females, equivalent to male prevalence of 60%, agreeing with the literature.^16^, ^17^ The age group consisted of individuals with a mean age of 66.92 years, agreeing with previously developed studies in which their characterization obtained patients with a mean age over 60 years and highlighted the intense dissatisfaction generated by tinnitus in this age group, leading to the effects of sleep and emotional problems.^18^, ^19^, ^20^, ^21^ A study pointed out that the prevalence of tinnitus is increased in individuals over 65years of age.^4^

The psychoacoustic measures found were compatible with the complaints of the respective patients. The mean tinnitus frequency was 5.32 kHz for the RE and 5.25 kHz for the LE, corroborating with a study which presented the acute pitch prevalence in 80% of their sample.^22^ The mean intensity found for tinnitus was 2.12 dBSL for the RE and 1.95 dBSL for the EO. The MML found was 3.47 dBSL for the RE and 3.22 dBSL for the LE (Table 3). These values corroborate with the literature where they define an average of 5 to 10 dBSL above the auditory threshold in dBHL at the frequency stipulated previously to perform both measurements.^23^

The scientific community suggests THI as the key tool for the verification of the nuisance caused by tinnitus and evaluation of the benefit of the intervention.^13^, ^14^, ^15^, ^16^, ^17^, ^18^, ^19^, ^20^, ^21^, ^22^, ^23^, ^24^ The THI data obtained in the initial evaluation corresponded to values equivalent to a moderate degree of discomfort, with a mean of 57.75 points for total score and 28.80 for functional aspect, 17.45 for emotional aspect and 11.05 for catastrophic aspect. Although they are apparently “decreasing” scores, depending on the subscales, the numbers vary with the number of questions, as explained in the methodology. A study points out that despite the measurement of degrees of discomfort, there are different impacts on patients’ quality of life.^18^

The comparison of the THI in the final and initial evaluations exposes statistically significant differences with *p* < 0.001 for the total score and all the subscales studied (functional, emotional and catastrophic). The results of the sample indicate that the average moderate degree of discomfort was reduced to discreet nuisance. These positive results corroborate with several studies that have also proved the benefit provided by the acoustic therapy.^20^, ^23^, ^25^, ^26^, ^27^

The same interpretation about the degree of discomfort occurred in the VAS instrument, where it was also compared in the initial and final evaluations and showed a statistically significant difference, indicating a significant reduction in discomfort. The mean initial score was 7.92, and at the end of the study, the mean was reduced to 2.85.

Acoustic therapy is composed of the insertion of sound enrichment into the daily life of the patient in order to provide relief from the annoyance generated by tinnitus. The same can be done or not through specific auricular devices, as well as alternatives: water cascades, relaxation sounds, and music, among others. Several treatments are proposed for tinnitus management, however, the use of HA associated or not with SG is still the majority. Studies observed that three months were sufficient to obtain the apex of improvement of the complaint.^28^, ^29^, ^30^, ^31^ The improvement obtained is due to the occurrence of cortical plasticity induced by acoustic stimulation, as explained in previous studies.^32^, ^33^

The present study demonstrated the effectiveness of SG in the treatment of tinnitus. Its effective use combined with HA and associated with counseling, resulted in improvements in the annoyance of tinnitus and consequently, in the quality of life index of individuals. Studies corroborate these data, verifying in their studies that there is no superiority between the interventions (combined or isolated stimulation of the amplification).^20^, ^21^, ^22^, ^23^ However, to date, there is no evidence of the effectiveness of this intervention because of the need for objective evaluation, which is the focus of the present study.

As a result, it is feasible that the measures established for the intervention be verified, observing if they are appropriate to the established program. It is possible to tune in and check whether the stimulation is in the audible range or approaches the level of intensity and frequency required, according to the proposed approach to sound therapy.

With these measures, the practitioner can assure that the noise determined by the patient at the time of SG adaptation to stimulation is being effective, since some approaches suggest that the patient himself chose his acoustic stimulation and this factor, as well as several that permeate the theme, is very subjective. In addition, the measurements favor the optimization of data manipulation and results, as well as monitoring during the tinnitus management process. It also favors the relationship of the professional with the patient, demonstrating more tangible data for him, quantifying his treatment and his progress during the process of habituation. However, to date there are no studies in the literature that have performed the approach of the SG in an objective way, by means of measurement in real ear.

In the correlations, a significant statistical significance was found. When correlating the THI, VAS and acuphenometry variables, as shown in Table 2, Table 3, a higher correlation coefficient strength was observed in the psychoacoustic threshold for intensity, especially in the RE, considering that the higher the degree of discomfort measured in the THI and in all its subscales, possibly the greater the intensity of tinnitus in dBSL measured by acuphenometry. These data make it possible to infer that the stronger the perception of tinnitus intensity, the greater its restriction in the THI questionnaire.

The terms used hereinafter: “SG verification” and “SG curve” refer to the difference in acuphenometry value (frequency and intensity thresholds transcribed in SPL) and the apex of the curve generated by the in situ evaluation in the live mode “Of the sound generator”. For example, a psychoacoustic threshold of 60 SPL at 4 kHz with the peak of the generator curve at 50 SPL at 4 kHz, predicts a check of 10 SPL, which is used in the statistical calculation.

When correlating the variables verification of SG and acuphenometry, it was also possible to observe statistically significant results with strong and proportional correlation coefficient, especially in relation to the psychoacoustic frequency threshold (Table 4). This correlation suggests that the higher the frequency of tinnitus, the greater the difference between the acuphenometry and the SG in real ear curve. These data may be derived from the possible limitation of the HA, considering that the acute frequencies are notably not amplified in some device technologies, perhaps because of the acute noise generating a greater inconvenience, being more uncomfortable or inferring that it is a frequency region that gets habituation with less acoustic stimuli.

In this same analysis, a statistically significant value was observed for the MML, with a strong correlation coefficient and inversely proportional in the RE. This data suggests that the greater the difference between the SG curve and the acuphenometry (consequently lower stimulation), the lower the MML, because with the lower masking level, less stimulation is required (Table 4).

Regarding the correlation between the SG and THI verification variables, no significant correlation coefficients were found, and it was not possible to establish any relationship between the degrees of discomfort with the SG curve from the acoustic stimulus selection by the patient at the time of adaptation (Table 5). Regarding the VAS, there was a strong and inversely proportional correlation coefficient, suggesting that the lower the difference between the stimulation and acuphenometry (SG curve), the higher the VAS score. These data suggest that there is high stimulation at this point in which tinnitus is present due to high discomfort (Table 6).

Among the existing approaches regarding the use of SGs, it is important to note that the totality of the sample contemplated noises below the psychoacoustic threshold obtained by the acuphenometry, there being no mixing point or masking, suggesting that any effective acoustic stimulation is valid, without any need to specifically stimulate the point or region of the tinnitus.^34^ It should be emphasized that the guidelines must be carried out by the auditory health services themselves, maintaining the standardization of the protocol, so that there is a probability of replication and comparison of the data in later studies.

The large individual variability of patients tested in repeated evaluations and differences among patients allocated to a treatment group contribute to discrete general contributions in treatment, with the lack of reapplication of related findings in all studies. Although there are a number of guidelines for good practice in tinnitus management, several options have no scientific evidence of their efficacy, and most of the literature review on the subject states that “the findings are inconclusive”.^35^

There is a need to present scientific evidence on tinnitus treatments to assist professionals in decision making, therapeutic management of patients and development of clinical guidelines that guide interventional evaluations and approaches.^31^ In this first moment, the objective verification by means of the measurement in real ear favored the understanding that the stimulation provided by the SG was below the marking of the psychoacoustic thresholds obtained in acufenometry, being necessary to deepen this knowledge and its applicability from new studies.

Agreeing with the present study, the authors concluded that although tinnitus heterogeneity is widely recognized by clinicians, many common sound-based tinnitus treatments are applied with limited assessment of individual differences.^36^ A recent study points out that there seems to be few guidelines for stimulus selection and sound therapy.^12^

## Conclusions

The present study concluded that the audiological intervention with any level of sound stimulus is enough to obtain a positive prognosis in the medium term. Data that specifies that the verification of sound generator was effective at the real ear measurements are important at the evaluation and intervention of the complaint. In addition, it points out that the greater the tinnitus perception, the greater its severity; and the greater the nuisance, the higher the psychoacoustics thresholds of frequency and the minimum threshold of masking.

## Conflicts of interest

The authors declare no conflicts of interest.

## References

[1] Roberts L.E., Eggermont J., Caspary D.M., Shore S.E., Melcher J.R., Kaltenbach J.A. (2010). Ringing ears: the neuroscience of tinnitus. J Neurosci.

[2] Mckenna L., Handscomb L., Derek J., Hall D.A. (2014). A scientific cognitive-behavioral model of tinnitus: novel conceptualizations of tinnitus distress. Front Neurol.

[3] Handscomb L.E., Hall D.A., Shorter G.W., Hoare D.J. (2017). Positive and negative thinking in tinnitus: factor structure of the tinnitus cognitions questionnaire. Ear Hear.

[4] Oiticica J., Bittar R.S.M. (2015). Tinnitus prevalence in the city of São Paulo. Braz J Otorhinolaryngol.

[5] Shargorodsky J., Curhan G.C., Farwell W.R. (2010). Prevalence and characteristics of tinnitus among US adults. Am J Med.

[6] Langguth B., Kreuzer P.M., Kleinjung T., Ridder D.D. (2013). Tinnitus: causes and clinical management. Lancet Neurol.

[7] Noreña A.J. (2011). An integrative model of tinnitus based on a central gain controlling neural sensitivity. Neurosci Biobehav Rev.

[8] Kostek B., Poremski T. (2013). A new method for measuring the psychoacoustical properties of tinnitus. Diagn Pathol.

[9] Moffat G., Adjout K., Gallego S., Thai-Van H., Collet L., Noreña A.J. (2009). Effects of hearing aid fitting on the perceptual characteristics of tinnitus. Hear Res.

[10] Newman C.W., Sandridge S.A. (2012). A comparison of benefit and economic value between two sound therapy tinnitus management options. J Am Acad Audiol.

[11] Vernon J.A., Meikle M.B., Tyler R.S. (2000). Tinnitus handbook.

[12] Searchfield G.D., Durai M., Linford T. (2017). A state-of-the-art review: personalization of tinnitus sound therapy. Front Psychol.

[13] Ferreira P.E.A., Cunha F., Onishi E.T., Branco F.C.A., Ganança F.F. (2005). Tinnitus Handicap Inventory: adaptação cultural para o português brasileiro. Pró-Fono R Atual Cient.

[14] WHO, World Health Organization (2016). http://www.who.int/pbd/deafness/hearing_impairment_grades/en/.

[15] Burguetti F.A.R., Peloggia A.G., Carvallo R.M.M. (2004). Limiares de audibilidade em altas frequências em indivíduos com queixa de zumbido. Arq Int Otorrinolaringol.

[16] Martines F., Bentivegna D., Martines E., Sciacca V., Martincoglio G. (2010). Characteristics of tinnitus with or without hearing loss: clinical observations in Sicilian tinnitus patients. Auris Nasus Larynx.

[17] Cabreira A.F. (2016).

[18] Pinto P.C.L., Sanchez T.G., Tomita S. (2010). The impact of gender, age and hearing loss on tinnitus severity. Braz J Otorhinolaryngol.

[19] Ferreira L.M.B.M., Ramos Júnior A.N., Mendes E.P. (2009). Caracterização do zumbido em idosos e de possíveis transtornos relacionados. Braz J Otorhinolaryngol.

[20] Parazzini M., Del Bo L., Jastreboff M., Tognola G., Ravazzani P. (2011). Open ear hearing aids in tinnitus therapy: an efficacy comparison with sound generators. Int J Audiol.

[21] Gibrin P.C.D., Melo J.J., Marchiori L.L.M. (2013). Prevalência de queixa de zumbido e prováveis associações com perda auditiva, diabetes mellitus e hipertensão arterial em pessoas idosas. CoDAS.

[22] Urnau D., Tochetto T.M. (2011). Características do zumbido e da hiperacusia em indivíduos normo-ouvintes. Arq Int Otorrinolaringol.

[23] Santos G.M. (2013).

[24] Landgrebe M., Azevedo A., Baguley D., Bauer C., Cacace A., Coelho C. (2012). Methodological aspects of clinical trials in tinnitus: a proposal for an international standard. J Psychosom Res.

[25] Sweetow R.W., Sabes J.H. (2010). Effects of acoustical stimuli delivered through hearing aids on tinnitus. J Am Acad Audiol.

[26] Rocha A.V., Mondelli M.F.C.G. (2017). Sound generator associated with the counseling in the treatment of tinnitus: evaluation of the effectiveness. Braz J Otorhinolaryngol.

[27] Suzuki F.A.B., Suzuki F.A., Yonamine F.K., Onishi E.T., Penido N.O. (2016). Effectiveness of sound therapy in patients with tinnitus resistant to previous treatments: importance of adjustments. Braz J Otorhinolaryngol.

[28] Tyler R.S., Tyler R.S. (2006). Tinnitus treatment: clinical protocols.

[29] Ferrari G.M.S., Sanchez T.G., Pedalini E.B.A. (2007). Eficácia do molde aberto para o controle do zumbido. Braz J Othorhinolaryngol.

[30] Carrabba L., Coad G., Constantini M., Del Bo L., Dyrlund O., Searchfield G. (2009).

[31] Rocha A.V. (2015).

[32] Noreña A.J., Eggermont J.J. (2005). Enriched acoustic evironment after noise trauma reduces hearing loss and prevents cortical map reorganization. J Neurosci.

[33] McNeill C., Tavola-Vieira D., Alnafjan F., Searchfield G., Welch D. (2012). Tinnitus pitch, masking, and the effectiveness of hearing aids for tinnitus therapy. Int J Audiol.

[34] Schad M.L., McMillan G.P., Thielman E.J., Groon K., Morse-Fortier C., Martin J.L. (2018). Comparison of acoustic therapies for tinnitus suppression: a preliminary trial. Int J Audiol.

[35] Hall D.A. (2017). Designing clinical trials for assessing the effectiveness of interventions for tinnitus. Trends Hear.

[36] Hoare D.J., Searchfield G.D., El Refaie A., Henry J.A. (2014). Sound therapy for tinnitus management: practicable options. J Am Acad Audiol.

